# Effects of Sweet Cherry Polyphenols on Enhanced Osteoclastogenesis Associated With Childhood Obesity

**DOI:** 10.3389/fimmu.2019.01001

**Published:** 2019-05-03

**Authors:** Filomena Corbo, Giacomina Brunetti, Pasquale Crupi, Sara Bortolotti, Giuseppina Storlino, Laura Piacente, Alessia Carocci, Alessia Catalano, Gualtiero Milani, Graziana Colaianni, Silvia Colucci, Maria Grano, Carlo Franchini, Maria Lisa Clodoveo, Gabriele D'Amato, Maria Felicia Faienza

**Affiliations:** ^1^Department of Pharmacy-Drug science, University of Bari Aldo Moro, Bari, Italy; ^2^Section of Human Anatomy and Histology, Department of Basic and Medical Sciences, Neurosciences and Sense Organs, University of Bari Aldo Moro, Bari, Italy; ^3^CREA-VE, Council for Agricultural Research and Economics–Research Centre for Viticulture and Enology, Turi, Italy; ^4^Section of Human Anatomy and Histology, Department of Emergency and Organ Transplantation, University of Bari, Bari, Italy; ^5^Paediatric Unit, Department of Biomedical Sciences and Human Oncology, University of Bari Aldo Moro, Bari, Italy; ^6^Interdisciplinary Department of Medicine, University of Bari Aldo Moro, Bari, Italy; ^7^Neonatal Intensive Care Unit, Di Venere Hospital, Bari, Italy

**Keywords:** obesity, inflammation, polyphenols, sweet cherry, osteoclastogenesis, CD14+/CD16+ monocytes, osteoporosis, osteopenia

## Abstract

Childhood obesity is associated with the development of severe comorbidities, such as diabetes, cardiovascular diseases, and increased risk of osteopenia/osteoporosis and fractures. The status of low-grade inflammation associated to obesity can be reversed through an enhanced physical activity and by consumption of food enrich of anti-inflammatory compounds, such as omega-3 fatty acids and polyphenols. The aim of this study was to deepen the mechanisms of bone impairment in obese children and adolescents through the evaluation of the osteoclastogenic potential of peripheral blood mononuclear cells (PBMCs), and the assessment of the serum levels of RANKL and osteoprotegerin (OPG). Furthermore, we aimed to evaluate the *in vitro* effects of polyphenol cherry extracts on osteoclastogenesis, as possible dietary treatment to improve bone health in obese subjects. High RANKL levels were measured in obese with respect to controls (115.48 ± 35.20 pg/ml vs. 87.18 ± 17.82 pg/ml; *p* < 0.01), while OPG levels were significantly reduced in obese than controls (378.02 ± 61.15 pg/ml vs. 436.75 ± 95.53 pg/ml, respectively, *p* < 0.01). Lower Ad-SoS- and BTT *Z*-scores were measured in obese compared to controls (*p* < 0.05). A significant elevated number of multinucleated TRAP^+^ osteoclasts (OCs) were observed in the un-stimulated cultures of obese subjects compared to the controls. Interestingly, obese subjects displayed a higher percentage of CD14^+^/CD16^+^ than controls. Furthermore, in the mRNA extracts of obese subjects we detected a 2.5- and 2-fold increase of TNFα and RANKL transcripts compared to controls, respectively. Each extract of sweet cherries determined a dose-dependent reduction in the formation of multinucleated TRAP^+^ OCs. Consistently, 24 h treatment of obese PBMCs with sweet cherry extracts from the three cultivars resulted in a significant reduction of the expression of TNFα. In conclusion, the bone impairment in obese children and adolescents is sustained by a spontaneous osteoclastogenesis that can be inhibited *in vitro* by the polyphenol content of sweet cherries. Thus, our study opens future perspectives for the use of sweet cherry extracts, appropriately formulated as nutraceutical food, as preventive in healthy children and therapeutic in obese ones.

## Introduction

Childhood obesity is one of the major health problems in the western world. It is associated with severe co-morbidities including diabetes, cardiovascular diseases (1, 2), and bone loss, which can occur early in the life (3, 4). It has been reported that the incidence of bone fractures increases in overweight/obese children and adolescents (5). The relationship between childhood obesity and bone impairment has been deepened in animal models. Indeed, Shu et al., found that mice fed with high fat diet (HFD) showed bone loss mainly due to high osteoclastic bone resorption, which is mediated by the increase of pro-osteoclastogenic cytokines and pre-osteoclasts in the bone marrow microenvironment (6).

Osteoclasts (OCs) derive from monocyte precursors which fuse thank to macrophage colony-stimulating factor (MCSF) and receptor activator of nuclear factor kappa-B ligand (RANKL) and become multinucleated cells able to resorb bone. RANKL is mainly produced by cells of the osteoblastic lineage. However, in inflammation also immune cells represent also an important source of the inflammatory cytokines [revised in Dar et al. (7)]. Recently, it has also been reported that bone marrow adipocytes produce RANKL (8), whose action could be inhibited by Osteoprotegerin (OPG), the soluble decoy receptor of RANKL (7). Other cytokines could also support osteoclastogenesis together with RANKL (9), such as TNFα. High levels of this cytokine have been demonstrated in bone diseases as well as in obesity (10–13). This last condition is associated with high levels of pro-inflammatory cytokines, such as interleukins, adipokines, and chemokines, which contribute to the chronic low level of inflammation and oxidative stress which are responsible of the different co-morbidities related to obesity (14, 15). This status of chronic inflammation can be prevented or even reversed by the loss of body weight through a reduction of food intake and enhanced physical activity (16). It has been reported that physical activity directly or indirectly decreased inflammation (17–19). Moreover, eating foods rich in bioactive anti-inflammatory compounds, such as omega-3 fatty acids (FAs) and polyphenols, has been demonstrated to reduce inflammation (20, 21). In particular, the anti-obesity effects of polyphenol-rich diets have been associated to the property of polyphenols to interact with adipose tissues (pre-adipocytes, adipose stem cells, and immune cells).

Sweet cherries are a source of dietary phenolic compounds (~1,500 mg total phenols per kg fresh weight), including phenolic acids (hydroxycinnamic acids) and flavonoids (anthocyanins, flavan-3-ols and flavonols), which are known for their health benefits and important role in preventing several chronic diseases related to oxidative stress (22, 23). Moreover, they show a low glycemic index respect to other fruits and vegetables and represent a source of vitamins, especially vitamin C and minerals, such as potassium, phosphorus, calcium, and magnesium (24, 25).

Studies *in vitro* and *in vivo* have reported that sweet cherries have anti-inflammatory and anti-carcinogenic activity, and characteristics for prevention of cardiovascular disease and diabetes (26).

In the light of these evidences and of the increasing interest on the polyphenol effects on childhood obesity, the aim of this paper were: (a) to deepen the mechanisms of bone impairment in obese children and adolescents, through the evaluation of the serum levels of RANKL and OPG together with the osteoclastogenic potential of peripheral blood mononuclear cells (PBMCs), and (b) to evaluate *in vitro*, the effects of polyphenols from sweet cherry extracts on osteoclastogenesis, as possible dietary treatment to improve bone health in obesity.

## Materials and Methods

### Subjects

Twenty-five obese children with a mean age of 10.8 ± 2.6 years were enrolled at Endocrinology Unit of Pediatric Hospital Giovanni XXIII, University A. Moro of Bari. Inclusion criteria were body mass index (BMI) ≥95th percentile for age and sex. Exclusion criteria were: type 2 diabetes mellitus, secondary or syndromic forms of obesity, hypothyroidism, Cushing disease, viral hepatitis, metabolic or genetic liver diseases, ongoing therapies for chronic systemic diseases. The control group consisted of 21 normal weight healthy children matched for age and gender, recruited on a voluntary basis in the outpatient clinic, who referred to hospital for minor surgery or electrocardiographic record for minor trauma to head, limbs, or chest pain. All the enrolled patients signed an informed consent form. The local ethic committee approved the study. The study was conducted in accordance to the criteria of the declaration of Helsinki. All subjects were in good general health and were not taking drugs in the last 3 months. Serum levels of (25)OH-vitamin D, osteocalcin, calcium, phosphorus, RANKL, OPG, and alkaline phosphatase were measured as previously reported (10). Bone quality was assessed by QUS measurements, performed with a DBM Sonic 1200 bone profiler (Igea S.r.l., Carpi, MO, Italy) employing a sound frequency of 1.25 MHz, as previously described (27).

### Cells and Culture Conditions

PBMCs were isolated by centrifugation of peripheral blood samples over Histopaque 1077 density gradient (Sigma Chemical, St. Louis, MO), and cultured in α-MEM (Life Technologies, Paisley, UK) supplemented with 10% fetal bovine serum, 100 IU/ml penicillin, and 100 μg/ml streptomycin (Life Technologies, Inc. Ltd, Uxbridge, UK). To obtain fully differentiated human OCs, the PBMCs were cultured in the presence or absence of 25 ng/ml recombinant human MCSF and 30 ng/ml RANKL (R&D Systems, Minneapolis, MN) for about 20 days. In some experiments, PBMCs were also cultured in the presence of 75 and 100 μg/ml of polyphenol extracts from Giorgia, Bigarreau, and Ferrovia both for mRNA extraction (24 h), MTT assay (24 h) (28), and for osteoclastogenesis (about 20 days) evaluation. The concentrations of the polyphenol extracts were selected according to literature data (29) and calculated according to a previous *in vitro* study on the effect of quercetin-containing cherry extracts on HepG2 cells (30) by considering 440 dalton as the average molecular weight of the compounds in the extracts; then, they were prepared through vacuum drying of the extracts and re-suspension in a suitable medium for the biological assays. Mature OCs were identified as tartrate-resistant acid phosphatase-positive (TRAP) multinucleated cells (Sigma Aldrich, Milan, Italy) containing three or more nuclei. OC resorbing activity was demonstrated by plating the cells on multiwell slides (4 × 10^5^ cells/well) coated with a calcium phosphate film (Millenium Osteologic; Millenium Biologix Inc, Ontario, Canada). This system incorporates a resorbable artificial bone in the form of submicron calcium phosphate films. The photomicrographs were obtained using a Ellipse E400 microscope (Nikon, Tokyo, Japan) equipped with Nikon Plan Fluor 10 × /0.30 dicl. The microscope was connected with a Nikon digital camera DxM 1200; the acquisition software was Lucia G version 4.61 (build 0.64) for Nikon Italy.

### Flow Cytometry Analysis

Fresh peripheral blood samples from patients and controls were stained with PerCp-CD14 and FITC-CD16 antibodies (all Beckmann Coulter, Milan, Italy). Events were acquired using C6 flow cytometer (Becton Dickinson Immunocytometry System, Mountain View, CA, USA). The area of positivity was determined using an isotype-matched mAb, a total of 10^6^ events for each sample were acquired.

### RNA Isolation and Real Time-PCR Amplification

Freshly isolated PBMCs of patients and controls, PBMCs treated for 24 h with polyphenol extracts from sweet cherries as well as OCs cultured in the presence of polyphenol extracts from sweet cherries were subjected to mRNA extraction using spin columns (RNeasy, QIAGEN, Hilden, Germany), and reverse-transcription using iScript Reverse Transcription Supermix (Bio-Rad Laboratories, Hercules, CA). The resulting cDNA was amplified using the SsoFast EvaGreen Supermix (Bio-Rad Laboratories) using the Chromo4 Real-Time PCR Detection System (Bio-Rad Laboratories). The following primer pairs were used for the real-time PCR amplification: RANKL S: CGTTGGATCACAGCACAT, RANKL AS: GCTCCTCTTGGCCAGTC; TNFα S: ATCTACTCCCAGGTCCTC, TNFα AS: GATGCGGCTGATGGTGT; calcitonin receptor (CalcR) S: AACAATAGAGCCCAAGCCATTTC, CalcR AS: CCAGCACAGCCATCCATCC; Cathepsin K (Cath K) S: GGCTCAAGGTTCTGCTAC, Cath K AS: GCTTCCTGTGGGTCTTCTTCC; RANK S: CAGGATGCTCTCATTGGTCAG, RANK AS: AGAAAGGAGGTGTGGATTGC; GAPDH S: TCATCCCTGCCTCTACTG; AS: TGCTTCACCACCTTCTTG.

### Reagents and Standards for Chemical Procedures

Formic acid, LC-MS grade water and acetonitrile were purchased from J.T. Baker (Deventer, Holland). Furulic acid, cyanidin-3-O-glucoside chloride, cyanidin-3-O-rutinoside chloride, delphinidin-3-O-glucoside chloride, quercetin-3-O-rutinoside, quercetin-3-O-glucoside, kaempferol-3-O-glucoside, kaempferol-3-O-rutinoside, isorhamnetin-3-O-glucoside, (+)-catechin, (–)-epicatechin, procyanidins B1 and B2, and epicatechin gallate were purchased from Extrasynthese (Genay, France). Cyanidin-3-O-sophoroside chloride, quercetin-4′-O-glucoside, chlorogenic acid, neochlorogenic acid, and cynarin were purchased from Phytolab (Vestenbergsgreuth, Germany).

### Fruit Collection

Three sweet cherry cultivars (cv. Ferrovia, Bigarreau, and Giorgia) grown in Apulia region (Southern Italy) was used in this study. Samples were harvested at commercial maturity (1st decade of May−2nd decade of June), on the basis of total soluble solids (TSS), measured as °Brix using a portable refractometer (Atago PR32, Norfolk, Virginia, USA), and titratable acidity (TA) which was determined in the juice by titration with 0.1 N of NaOH (J.T. Baker, Deventer, Holland) to a pH 7 end point (TSS = ~ 17 °Brix; TA = ~ 7 g/L of citric acid equivalents), in 2014 season using 7 years-old sweet cherry trees located in Turi. The trees were trained to a central leader system and planted at a spacing of 4 m × 4 m and were grown under usual conditions of irrigation, fertilization, and pest control (31). Five kg of cherries for each variety were taken on the same day, from four different branches of an individual tree and mixed, then they were frozen in liquid nitrogen and vacuum packed in plastic bags and stored at −80°C for further analysis.

### Extraction of Polyphenols From Sweet Cherry and HPLC-MS/MS Analysis

Polyphenols were extracted from cherries and analyzed through a capillary HPLC 1100 coupled with a triple quadrupole QQQ mass detector (Agilent Technologies Palo Alto, CA, U.S.A.), following the procedure proposed in our previous researches (31, 32).

Roughly 100 g of partially defrosted sweet cherry sample were pitted and a homogenate was obtained using an IKA A11—basic homogenizer (IKA—WERKE GMBH & CO.KG—Germany). To avoid compounds degradation, the homogenization was completed in darkness and the sample was placed on ice during the whole procedure (around 5 min). Ten gram of homogenate was put in a glass flask with 10 mL of 1% hydroxybutyl anisole (BHA) in methanol and 100 μL of ferulic acid internal standard solution (1,000 μg/mL of methanol). Then, the obtained solution was sonicated in an ultrasonic bath of 130 W and 40 kHz (SONICA 2200 EP, SOLTEC, Milano, Italy) for 1 h at 25°C and the liquid phase was separated by filtration under vacuum. The extraction procedure was repeated twice for the solid phase utilizing fresh methanol (10 and 5 mL for 30 min, respectively). Finally, the pooled extracts were concentrated down to 10 mL through a rotavapor Buchi-R-205 under vacuum at 40°C, and stored at −25°C until further analysis.

A Zorbax column SC-C18 (50 × 2.1 mm i.d., particle size 1.8 μm, Agilent Technologies) was used, with the following gradient system: water/formic acid (99:1, v/v) (solvent A) and acetonitrile/formic acid (99:1, v/v) (solvent B), 0.8 min, 95% A−5% B; 2.1 min, 90% A−10% B; 5.6 min, 88% A−12% B; 8 min, 81% A−19% B; 9.2 min 81% A−12% B; 11.2 min 5% A−95% B; 12.8 min 5% A−95%; 13.2 min 95% A−5%; stop time 15 min. The column was kept at 60°C, the flow was maintained at 0.5 mL/min and the sample injection was 1.1 μL. Both positive and negative ESI mode was used for ionization of molecules with capillary voltage at 4,000 V. Nitrogen was used both as drying gas at a flow rate of 8 L/min and as nebulizing gas at a pressure of 30 psi. Temperature of drying gas was 350**°**C. In the full scan (MS) and product ion (MS/MS) modes, the monitored mass range was from *m/z* 100 to 1,200. Typically, 2 runs were performed during the HPLC-ESI-MS analysis of each sample. First, an MS full-scan acquisition was performed to obtain preliminary information on the predominant *m/z* ratios observed during the elution. An MS/MS full-scan acquisition was then performed: Quadrupole 1 filtered the calculated *m/z* of each compound of interest, while Quadrupole 3 scanned for ions produced by nitrogen collision of these ionized compounds in the chosen range at a scan time of 500 ms/cycle. All data were acquired and processed using Mass Hunter software (version B.01.04; Agilent Technologies). The optimized parameters (fragmentor voltage and collision energy) for each compound together with the mass transitions adopted for multiple reaction monitoring (MRM) are listed in Table 1S (Supporting Information). To gauge linearity, calibration curves with five/seven concentration points for each compound were prepared separately. Calibration was performed by linear regression of peak-area ratios of the polyphenols to the relative internal standard vs. the respective standard concentration.

### Statistical Analyses

Means and standard deviations of the raw data and regression analysis of calibration samples were carried out using STATISTICA 6.0 software package (StatSoft Inc., Tulsa, OK, U.S.A.).

For statistical analyses of clinical data, the Statistical Package for the Social Sciences (SPSS) for Windows, version 22.0 (SPSS Inc., Chicago, IL, USA) was used. Comparison between groups were performed by *T*-test. Correlations were analyzed with Spearman or Pearson correlation test. The limit of statistical significance was set at 0.05.

## Results

### Clinical Characteristics

The characteristics of the study population were reported in Table 1. Although, in the normal range, lower Ad-SoS- and BTT- *Z*-scores were measured in obese patients compared to controls (*P* < 0.05). The serum levels of 25-OH Vitamin D, calcium, phosphorus, and osteocalcin were comparable to those measured in controls. Interestingly, higher RANKL levels were measured in obese patients with respect to the controls (115.48 ± 35.20 pg/ml vs. 87.18 ± 17.82 pg/ml; *p* < 0.01), while OPG levels were significantly reduced in obese patients than in controls (378.02 ± 61.15 pg/ml vs. 436.75 ± 95.53 pg/ml, respectively, *p* < 0.01). With adjustment for age RANKL levels correlated with waist circumference (*r* = 0.144 *p* < 0.022), and SDS-BMI (*r* = 0.129 *p* < 0.038), whereas OPG levels correlated with waist circumference (*r* = −0.348 *p* < 0.0001), SDS-BMI (*r* = −0.381 *p* < 0.0001), BTT-*Z*-score (*r* = 0.208 *p* < 0.002), HOMA-IR (*r* = −0.359 *p* < 0.0001).

**Table 1:** Characteristics of study population.

	**Controls *N* = 21**	**Obese patients *N* = 25**
Gender (male/female)	9/12	9/16
Age (yr)	8.23 ± 3.19	10.8 ± 2.6
Tanner Stage (I, II, III, IV, V)	6,10,4,1,0	8,11,4,2,0
Height SDS	0.36 ± 1.02	0.23 ± 1.48
Weight SDS	0.43 ± 0.87	2.22 ± 0.70^**^
BMI-SDS	0.25 ± 0.78	2.31 ± 0.41^**^
Waist circumference (cm)	72.5 ± 7.2	92.04 ± 23.08^**^
Total cholesterol (mg/dl)	154.8 ± 28.32	164 ± 33.18
HDL (mg/dl)	55.67 ± 9.30	49.08 ± 8.13
LDL (mg/dl)	97.10 ± 21.03	107.54 ± 37.62
Triglycerides (mg/dl)	67.24 ± 19.16	73.00 ± 32.74
Insulin (microU/mL)	9.78 ± 4.50	24.60 ± 12.02^**^
Glucose (ml/dl)	81.16 ± 7.14	87.22 ± 11.35
HOMA-IR	2.56 ± 0.40	4.93 ± 1.91^**^
25-OH Vitamin D (ng/ml)	38.64 ± 14.70	29.70 ± 12.89
Osteocalcin (ng/ml)	38.26 ± 19.22	47.44 ± 21.02
PTH (pg/ml)	43.05 ± 15.06	44.07 ± 17.09
Calcium (mg/dl)	9.71 ± 0.40	9.43 ± 0.41
Phosphorus (mg/dl)	4.54 ± 1.4	4.54 ± 0.51
Ad-Sos-Z-score	0.48 ± 0.85	−1.05 ± 1.17^*^
BTT-Z-score	0.15 ± 0.72	−0.39 ± 1.23^*^
RANKL (pg/ml)	87.18 ± 17.82	115.48 ± 35.20§
OPG (pg/ml)	436.75 ± 95.53	378.02 ± 61.15§

SDS, standard deviation score; BMI, body mass index; PTH, parathyroid hormone; Ca, calcium; P, phosphorus; B-ALP, bone alkaline phosphatase; RANKL, receptor activator of nuclear factor kappa-B ligand; OPG, osteoprotegerin. §p < 0.01;

* p < 0.05;

** p < 0.001.

### Osteoclastogenesis in Obese Children and Adolescents

**Figure 1:**
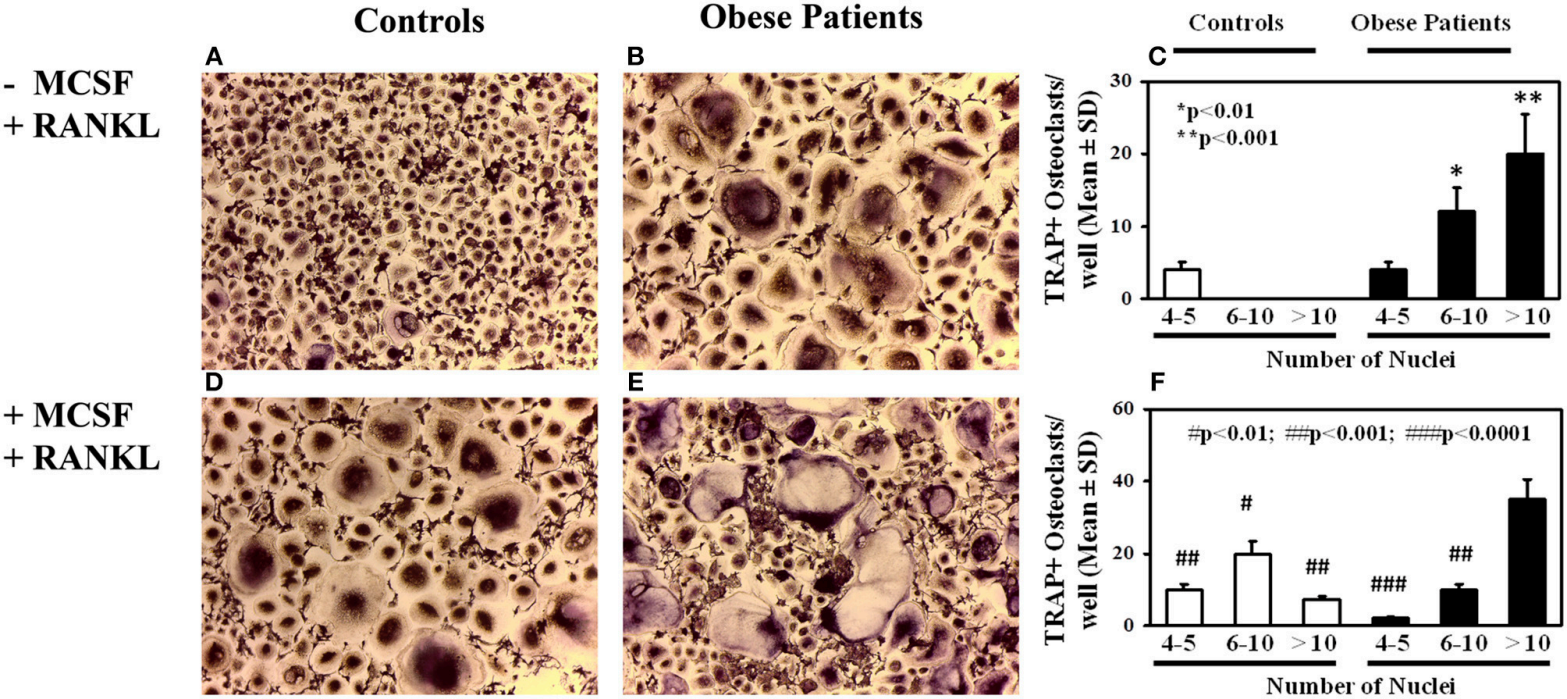
Osteoclastogenesis in obese subjects. Osteoclasts (OCs) identified as tartrate-resistant acid phosphatase-positive (TRAP^+^) and multinucleated cells with three or more nuclei, differentiated from peripheral blood mononuclear cells (PBMCs) of obese subjects and controls. Few small OCs differentiated in un-stimulated PBMC cultures of a representative control **(A)**, whereas multinucleated TRAP^+^ OCs differentiated from un-stimulated PBMCs from a representative obese subject **(B)**. The histogram includes OC count deriving from all subjects' cultures, stratified according the number of nuclei per OC **(C)**. In PBMC cultures from the controls, OCs differentiate following MCSF and RANKL addition **(D)**, otherwise in cultures from obese subjects growth factor addition did not further increase osteoclastogenesis **(E)**, compared with the un-stimulated cultures. The histogram reports the results deriving from all the enrolled subjects **(F)**.

OC formation was evaluated in cultures of PBMCs from obese patients and controls. A significant elevated number of multinucleated TRAP^+^ OCs were counted in the un-stimulated cultures of obese patients (Figure 1B) compared to the controls (Figure 1A), as reported in the histogram (Figure 1C). The addition of the pro-osteoclastogenic M-CSF and RANKL in the cultures from patients did not affect the OC number, but they appear larger compared those observed in the un-stimulated cultures (Figure 1E). Indeed, the number of large OCs (>10 nuclei) was greater in stimulated compared with un-stimulated cultures from obese patients (35 ± 5 vs. 20 ± 6, *p* < 0.01). Conversely, M-CSF and RANKL are necessary to trigger OC formation in cultures from controls (Figure 1D), as reported in the histogram (Figure 1F).

**Figure 2:**
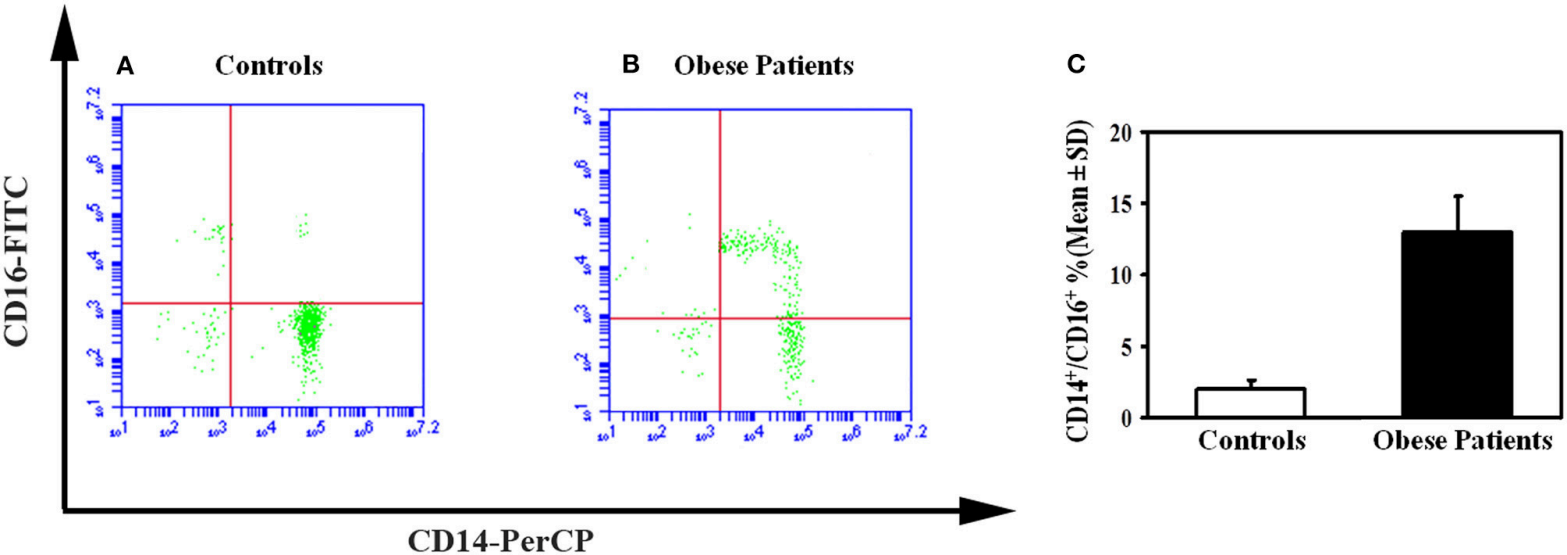
Circulating osteoclast precursors in obese subjects. A representative flow cytometry dot plots displayed the percentage of circulating osteoclast precursors, identified as CD14^+^/16^+^ cells in control **(A)** and obese subjects **(B)**. The histograms represent the percentage of CD14^+^/CD16^+^ cells measured for all enrolled obese and control subjects by flow cytometry **(C)**.

**Figure 3:**
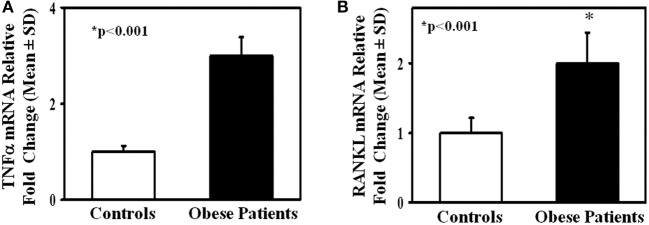
TNFα and RANKL expression in lymphomonocytes from obese subjects. mRNA levels of TNFα **(A)** and RANKL **(B)** in lymphomonocytes from all enrolled controls and obese subjects. Obese subjects expressed higher levels of TNFα and RANKL compared to the controls.

To investigate the mechanisms of the enhanced osteoclastogenesis in obese we evaluated both the percentage of CD14^+^/CD16^+^ circulating pre-osteoclasts as well as the levels of the pro-osteoclastogenic cytokines RANKL and TNFα in PBMC extracts. Interestingly, patients displayed a high percentage of CD14^+^/CD16^+^, compared to the controls (Figure 2). Furthermore, in mRNA extracts of obese patients we detected a 2.5- and 2-fold increase of TNFα and RANKL transcripts compared to controls, respectively (Figure 3).

### Effect of Polyphenols From Sweet Cherry on the Spontaneous Osteoclastogenesis of Obese Children and Adolescents

Interestingly, we also evaluated *in vitro* the effect of polyphenol cherry extracts on osteoclastogenesis as possible dietary treatment to improve bone health in obesity.

#### Polyphenols Content in the Cherries Extracts

Table 1S listed the amount of the main flavonoids (anthocyanins, flavan-3-ols, and flavonols) and chlorogenic acids, which were identified as previously described (31, 32), quantified by HPLC-MS/MS analyses in the tested cherries extracts. The content of the phenolic compounds appeared slightly lesser in the extract of Giorgia (1,391 mg/100 g FW) than Bigarreu and Ferrovia (1,820 and 1,768 mg/100 g FW, respectively), even though both the three varieties were principally characterized by anthocyanins, especially cyanidin-3O-rutinoside, accounting for 19–30% of total polyphenols, and chlorogenic acids (particularly, *trans*-3-O-coumaroylquinic acid and *trans*-3-O-caffeoylquinic acid) ranging between 70 and 80% of the total polyphenols (Table 1S).

#### Polyphenols Effect on Osteoclastogenesis of Obese Children and Adolescents

**Figure 4:**
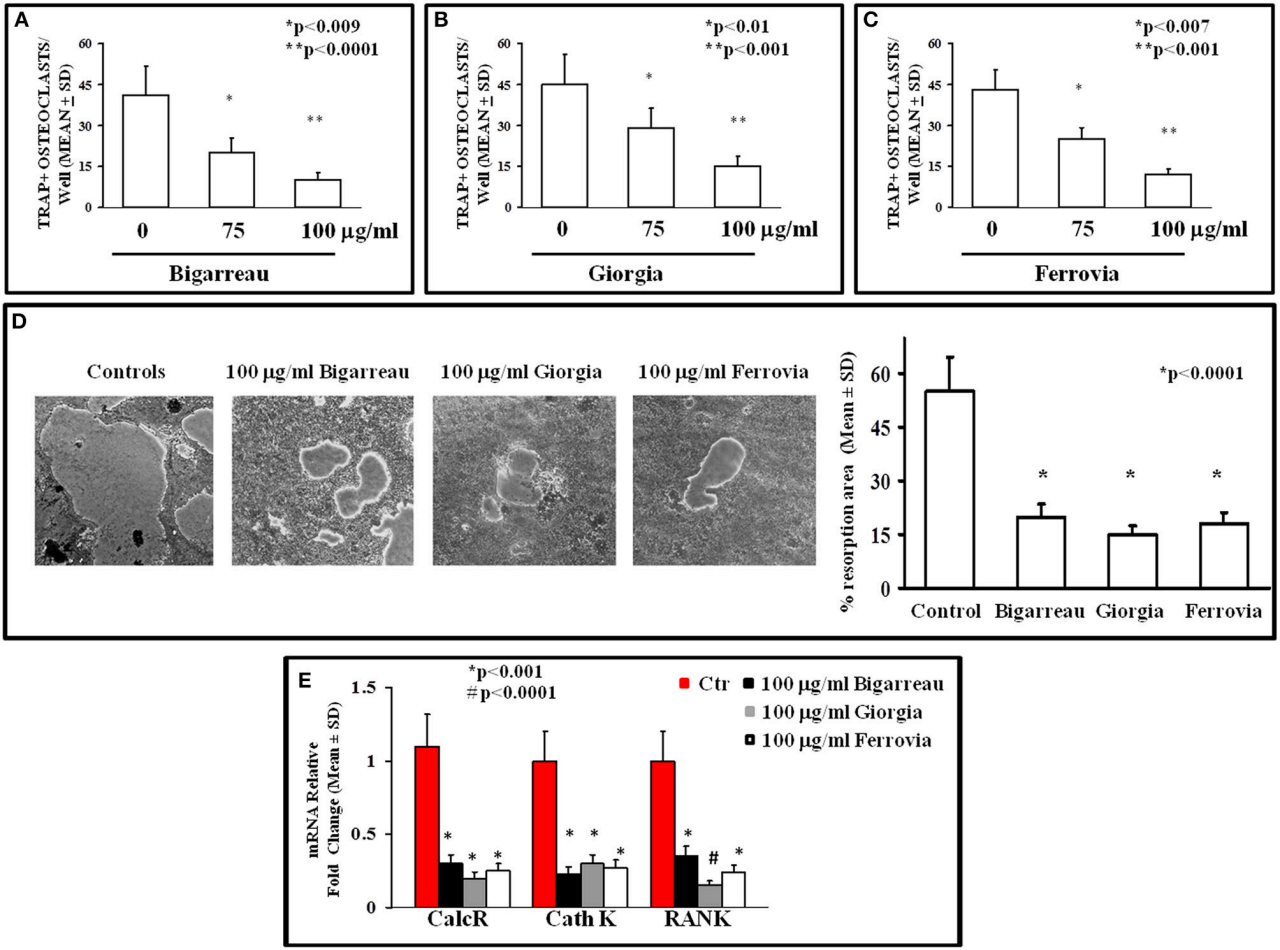
Osteoclastogenesis inhibition by polyphenol extracts from Giorgia, Bigarreau, and Ferrovia. The formation of multinucleated TRAP^+^ OCs was evaluated in un-stimulated PBMCs from all obese patients cultured in the absence or presence of 75 and 100 μg/ml polyphenol extracts from Bigarreau **(A)**, Giorgia **(B)**, and Ferrovia **(C)**. PBMCs from the patients, cultured on Millenium slides coated with a calcium phosphate film, formed large resorption areas, that were reduced following the treatment with 100 μg/ml polyphenol extracts from Bigarreau, Giorgia, and Ferrovia, as quantified in the histogram **(D)**. The mRNA levels of calcitonin receptor (CalcR), cathepsin K (Cath K), and RANK was evaluated in PBMCs from obese patients cultured in the absence or presence of 100 μg/ml polyphenol extracts from Bigarreau, Giorgia, and Ferrovia **(E)**.

**Figure 5:**
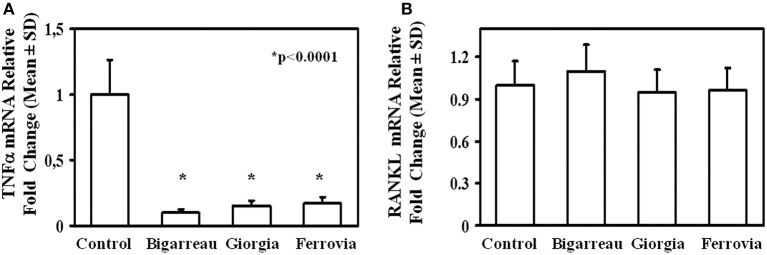
RANKL and TNFα expression in lymphomonocytes from obese subjects. Twenty-four hours treatment of PBMCs from obese subjects with 100 μg/ml polyphenol extracts from Giorgia, Bigarreau, and Ferrovia resulted in a significant reduction of the mRNA levels of TNFα **(A)**, whereas RANKL levels were unchanged **(B)**. The results are referred to all obese subjects.

**Figure 6:**
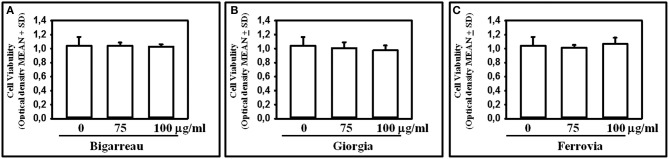
Effect of Polyphenol extracts from Giorgia, Bigarreau, and Ferrovia on PBMC viability. PBMCs were treated for 24 h with 75 and 100 μg/ml polyphenol extracts from Bigarreau **(A)**, Giorgia **(B)**, and Ferrovia **(C)** and analyzed by MTT assay to evaluate cell viability. Results are expressed as mean values of optical density at 570 nm ± standard deviation (SD).

We investigated the effect of polyphenol extracts from Giorgia, Bigarreau, and Ferrovia on PBMC cultures of patients. We demonstrated that each extract determined a dose-dependent reduction in the formation of multinucleated TRAP^+^ OCs (Figures 4A–C). Furthermore, using the highest dose of polyphenol extracts from Giorgia, Bigarreau, and Ferrovia we demonstrated that the treatment also resulted in a significant reduction of resorption activity (Figure 4D), together with a significant reduction of the expression of OC marker genes, such as calcitonin receptor, cathepsin K and RANK (Figure 4E). Consistently, 24 h treatment of PBMCs from patients with polyphenol extracts from Giorgia, Bigarreau, and Ferrovia resulted in a significant reduction of the expression of TNFα (Figure 5A), whereas RANKL levels were unchanged (Figure 5B). Furthermore, by MTT we demonstrated that polyphenol extracts did not significantly affect cell viability of PBMCs from patients (Figure 6). These results suggested that polyphenols from sweet cherry inhibit osteoclastogenesis through the reduction of pro-osteoclastogenic cytokines, without affecting cell viability.

## Discussion

This study demonstrated that in obese children the reduced bone mineral density (BMD) is associated to the decrease of OPG levels, the increase of RANKL levels, enhanced formation of OCs, of circulating pre-osteoclasts, and pro-osteoclastogenic cytokines. Interestingly, the spontaneous osteoclastogenesis is inhibited *in vitro* by sweet cherry polyphenol extracts.

Previous studies demonstrated that obese subjects showed significantly lower OPG levels respect to the controls (33–35); however no correlation has been reported between OPG and BMI (36, 37). Otherwise, few studies measured higher levels of OPG in obese subjects compared with the controls (38, 39). However, all the previous studies correlated the levels of OPG with the altered HOMA-IR, fasting insulin or glucose. Our study, to our knowledge, is the first demonstrating a direct correlation between OPG levels and BTT-Z score in obese children.

It is known that obesity is associated with bone fragility and the reduced OPG levels could contribute to this status. We also found increased RANKL levels which could explain the bone impairment associated with excess of adipose tissue. Interestingly, we found that RANKL levels positively correlated with waist circumference. The correlation between a central obesity parameter, as the waist circumference, and RANKL levels detected in serum and saliva samples has been previously demonstrated (40). Our data confirmed that visceral fat accumulation represents the main parameter which can predict the entity of bone impairment in obese subjects. These findings also suggest to evaluate bone status in obese subjects with a higher waist circumference than normal values. It is known that RANKL and OPG altered levels have been associated to the altered osteoclastogenesis characterizing bone diseases (41–43). Indeed, it has been demonstrated that anti-RANKL antibody is useful in the treatment of osteoporosis (44). The alterations of OPG and RANKL levels together with the increase of CD14^+^/CD16^+^ circulating pre-osteoclasts and TNFα levels are consistent with the spontaneous osteoclastogenesis of our obese patients as well as of other inflammatory diseases associated with bone loss (45). CD14^+^/CD16^+^ cells have been linked with erosive bone diseases, such as psoriatic arthritis and multiple myeloma (46–48). It is known that CD14^+^/CD16^+^ cells display an enhanced pro-osteoclastogenic activity (47, 48) thus supporting the key role of this cells in the alteration of bone health in obesity. Consistently, rodent models of obesity also demonstrated the increase of OC precursors in the bone marrow (49). Consistently, the ongoing theory sustains that weight gain determines local inflammation that stimulate the increased recruitment of circulating pro-inflammatory (Ly6C^hi^) monocytes, also capable of differentiate in OCs in bone. Recruited monocytes differentiate into an M1 macrophage phenotype which is responsible of the chronic inflammation and thus organ damage associated to obesity (15).

An increased mRNA levels of pro-osteoclastogenic molecules such as RANKL and TNFα has been found in young mice fed with HFD (6). Interestingly, our results also displayed high mRNA levels of TNFα and RANKL in PBMCs from obese subjects. It has been reported that childhood obesity is associated to a state of chronic low-grade inflammation as well as numerous inflammation-related molecules such as TNFα, interleukin 6 (IL-6), and leptin. High levels of these molecules have been linked to co-morbidities associated to obesity (50–53). Furthermore, consisting with our results, transgenic mouse expressing human TNFα determines the augment of OC precursor percentage (54).

As countermeasure against chronic low-grade inflammation associated to obesity is represented by dietary advice and nutraceuticals (55). Evidences from *in vitro* and experimental models suggest the effects of polyphenols on obesity, obesity-related inflammation, and other metabolic disorders. Their effects include: to induce satiety, to stimulate energy expenditure by inducing thermogenesis in brown adipose tissue, to inhibit adipocyte differentiation and promote adipocyte apoptosis, to modulate lipolysis and activate oxidation (56). Evidence for the effects of polyphenols on obesity and weight control in adult subjects is inconsistent due to the heterogeneity among study populations, intervention period, and polyphenol supplements (57). At the best of our knowledge, there are no studies about the effects of polyphenols extracts on childhood obesity and its comorbidities.

The innovative aspect of this study is related to the inhibition of the spontaneous osteoclastogenesis and reduction of TNFα mRNA levels in PBMC cultures from obese children with the use of polyphenol-rich cherry extracts. This inhibitory effect has been observed with all the three cultivars of sweet cherries, although the content of the phenolic compounds appeared slightly lesser in the extract of Giorgia than Bigarreu and Ferrovia, even though both the three varieties were principally characterized by anthocyanins, especially cyanidin-3O-rutinoside, and chlorogenic acids. These polyphenols' compounds play an important role as antioxidants for bone health, both in young people, in order to favor the formation of peak bone mass, and in the elderly and in menopausal women in order to prevent bone loss. Moreover, the use of these antioxidant compounds has been proposed in anti-resorption therapies considering also that they are able to reduce the OC activity without determining their apoptosis, which is useful to restore physiological bone remodeling (58). Consistently, it has been reported that tea and dried plum polyphenols *in vitro* inhibited osteoclastogenesis (29, 59). Of note, it has also been demonstrated the inhibitory effects of sweet cherry anthocyanins on obesity development in HFD fed mice, by slowing down TNFα and IL-6 levels (60). However, this study did not evaluate the effect on bone, which is known to be negatively affected by obesity as well as by HFD. Conversely, Shen et al., reported that in rats green tea polyphenols improved bone health in HFD-induced obesity by the suppression of bone cell activity (61, 62). Although the positive effect of green tea administration in obese patients has been evaluated in different studies [revised in Suzuki et al. (63)], there were not published data on bone effects. These literature reports together with our findings let us to speculate that also sweet cherry polyphenols can have a protective effect on bone both in HFD fed mice and obese patients.

## Conclusions

Our study, to our knowledge, is the first demonstrating in obese children a spontaneous osteoclastogenesis inhibited by polyphenols from sweet cherry extracts, through the reduction of TNFα, without affecting cell viability. We also demonstrated that the spontaneous osteoclastogenesis observed in PBMCs from obese children is supported by the high percentage of circulating CD14^+^/CD16^+^ cells and the elevated levels of RANKL and TNFα. Our study opens future perspectives for the use of cherry extracts, appropriately formulated as nutraceuticals as preventive in healthy children and therapeutic in obese ones.

## Data Availability

The datasets generated for this study are available on request to the corresponding author.

## Ethics Statement

All the enrolled patients signed an informed consent form. The local ethic committee approved the study. The study was conducted in accordance to the criteria of the declaration of Helsinki.

## Author Contributions

GB, MF, and FC developed the concept and designed the experiments. GC performed most experiments and analyzed data. LP performed cell cultures and ELISA. SB and GS performed flow cytometry. MF and GD provided patients' samples and clinical data. FC, ACi, ACo, GM, MC, PC, and CF developed the chemical part of the paper. GB, MF, FC, and MC wrote the manuscript and all other authors commented on the manuscript.

### Conflict of Interest Statement

The authors declare that the research was conducted in the absence of any commercial or financial relationships that could be construed as a potential conflict of interest.
