# Supplementary material for: Effects of Sweet Cherry Polyphenols on Enhanced Osteoclastogenesis Associated With Childhood Obesity
Source: Front Immunol. 2019 May 3;10:1001. doi: 10.3389/fimmu.2019.01001 (PMC6509551; doi:10.3389/fimmu.2019.01001)
Supplement: Supplementary file 1 [file Table_1.doc]

**Table S1.** Acquisition parameters for MRM UHPLC-MS/MS analyses.

| **Compound** | **Q_1_ (*m/z*)** | **Q_3_ (*m/z*)** | **Frag (V)** | **CE (V)** | **Polarity** |
| --- | --- | --- | --- | --- | --- |
| Anthocyanins |  |  |  |  |  |
| Delphinidin-3-O-glucoside (ISTD) | 465 | 303 | 140 | 20 | positive |
| Cyanidin-3-O-sophoroside | 611 | 287 | 140 | 20 | positive |
| Cyanidin-3-O-glucoside | 449 | 287 | 140 | 20 | positive |
| Cyanidin-3-O-rutinoside | 595 | 287 | 140 | 20 | positive |
| Flavan-3-ols |  |  |  |  |  |
| Procyanidin B_1_ | 577 | 425 | 140 | 10 | negative |
| (+)-Catechin | 289 | 245 | 120 | 20 | negative |
| Procyanidin B_2_ | 577 | 425 | 140 | 10 | negative |
| (-)-Epicatechin | 289 | 245 | 120 | 20 | negative |
| Epicatechin-gallate (ISTD) | 441 | 169 | 120 | 20 | negative |
| Flavonols |  |  |  |  |  |
| Quercetin-3-O-rutinoside | 609 | 301 | 140 | 35 | negative |
| Quercetin-3-O-glucoside | 463 | 301 | 140 | 20 | negative |
| Kaempferol-3-O-glucoside | 447 | 284 | 140 | 20 | negative |
| Kaempferol-3-O-rutinoside | 593 | 285 | 140 | 30 | negative |
| Quercetin-4’-O-glucoside | 463 | 301 | 140 | 15 | negative |
| Isorhamnetin-3-O-glucoside (ISTD) | 477 | 314 | 140 | 20 | negative |
| Chlorogenic acids |  |  |  |  |  |
| 3-O-(4’-O-caffeoyl glycosyl)-quinic acid | 515 | 353 | 100 | 10 | negative |
| *cis*-3-O-caffeoyl-quinic acid | 353 | 191 | 80 | 10 | negative |
| 5-O-(4’-O-caffeoyl glycosyl)-quinic acid | 515 | 353 | 100 | 10 | negative |
| *trans-*3-O-caffeoyl-quinic acid  (neochlorogenic acid) | 353 | 191 | 80 | 10 | negative |
| *cis*-3-O-coumaroyl quinic acid | 337 | 163 | 80 | 10 | negative |
| *trans*-3-O-coumaroyl quinic acid | 337 | 163 | 80 | 10 | negative |
| 1,3-dicaffeoylquinic acid  (cynarin) | 515 | 353 | 100 | 10 | negative |
| *trans*-5-O-caffeoylquinic acid  (chlorogenic acid) | 353 | 191 | 80 | 10 | negative |
| 4-O-caffeoyl-quinic acid  (cryptochlorogenic acid) | 353 | 191 | 80 | 10 | negative |
| 4-O-(4’-O-caffeoyl glycosyl)-quinic acid | 515 | 341 | 100 | 10 | negative |
| ferulic acid (ISTD) | 193 | 134 | 80 | 10 | negative |
| methyl 3-O-caffeoylquinate | 367 | 161 | 80 | 10 | negative |
| *cis*-4-O-coumaroylquinic acid | 337 | 173 | 80 | 10 | negative |
| *cis*-5-O-caffeoylquinic acid | 353 | 191 | 80 | 10 | negative |
| *trans*-4-O-coumaroylquinic acid | 337 | 173 | 80 | 10 | negative |
| methyl coumaroylquinate I | 351 | 145 | 80 | 10 | negative |
| methyl coumaroylquinate II | 351 | 145 | 80 | 10 | negative |
| methyl 4-O-caffeoylquinate | 367 | 161 | 80 | 10 | negative |
| methyl coumaroylquinate III | 351 | 145 | 80 | 10 | negative |
| methyl 5-O-caffeoylquinate | 367 | 161 | 80 | 10 | negative |
| methyl coumaroylquinate IV | 351 | 145 | 80 | 10 | negative |
| di-*trans*-3,5-di-O-caffeoylquinic acid | 515 | 353 | 100 | 10 | negative |
| *cis*,*trans*-3,5-di-O-caffeoylquinic acid | 515 | 353 | 100 | 10 | negative |
| 3-O-coumaroyl-5-O-caffeoylquinic acid | 499 | 337 | 100 | 10 | negative |
| 3-O-caffeoyl-5-O-coumaroylquinic acid | 499 | 353 | 100 | 10 | negative |
| 3-O-coumaroyl-4-O-caffeoylquinic acid | 499 | 337 | 100 | 10 | negative |

MRM, multiple reaction monitoring; Q_1_, precursor ion mass; Q_3_, product ion mass; Frag, fragmentor voltage; CE, collision energy; ISTD, internal standard.
